# Molecular Prevalence and Genetic Diversity of *Blastocystis* sp. in Slaughtered Ruminants in Qazvin Province, Iran: A Zoonotic Concern

**DOI:** 10.1002/vms3.70898

**Published:** 2026-04-21

**Authors:** Milad Badri, Fariba Najar Hoseini, Mohammadreza Mohammadi, Aida Vafae Eslahi, Amin Karampour, Faezeh Mohammadi, Meysam Olfatifar, Daniel Diaz, Ali Asghari

**Affiliations:** ^1^ Medical Microbiology Research Center Qazvin University of Medical Sciences Qazvin Iran; ^2^ Department of Medical Parasitology and Mycology School of Medicine Qazvin University of Medical Sciences Qazvin Iran; ^3^ Gastroenterology and Hepatology Diseases Research Center Qom University of Medical Sciences Qom Iran; ^4^ Facultad de Ciencias Universidad Nacional Autónoma de México Ciudad de México México; ^5^ Department of Basic Medical Sciences Khoy University of Medical Sciences Khoy Iran

**Keywords:** *Blastocystis* sp, Iran, prevalence, Qazvin, ruminants, subtypes

## Abstract

**Background:**

*Blastocystis* sp. is a genetically diverse intestinal protist with global distribution in humans and animals. Despite increasing evidence of its zoonotic potential, data on its prevalence and subtype (ST) diversity in Iranian ruminants remain limited. This study aimed to investigate the molecular prevalence and ST distribution of *Blastocystis* sp. in cattle, sheep and goats in Qazvin Province, Iran.

**Methods:**

A total of 142 faecal samples were collected from slaughterhouses in Abyek, Buin Zahra and Takestan counties in 2025, including 84 cattle, 26 sheep and 32 goats. Genomic DNA was extracted using the QIAamp DNA Stool Mini Kit. PCR amplification was performed based on the SSU rRNA gene, and products were visualized on 1.5% agarose gels. Positive amplicons were randomly selected for sequencing, and STs were assigned by BLAST comparison with GenBank references. Phylogenetic relationships were constructed using MEGA X with the Neighbour‐Joining method. Statistical associations between infection and host variables were evaluated using chi‐square and Fisher's exact tests.

**Results:**

*Blastocystis* sp. DNA was detected in 32 of 142 ruminant samples (22.5%). Prevalence was 19% (16/84) in cattle, 27.3% (7/26) in sheep and 28.1% (9/32) in goats, with no significant difference among species (*p* = 0.486). Infection was significantly higher in females (30.1%) than in males (14.5%) (*p* = 0.026). Further analysis revealed no significant correlation between infection status and age group (*p* = 0.454) or slaughterhouse location (*p* = 0.698). Sequencing 16 randomly selected PCR‐positive isolates revealed five zoonotic STs: ST1 (*n* = 2), ST5 (*n* = 3), ST7 (*n* = 1), ST10 (*n* = 9) and ST14 (*n* = 1). ST10 and ST14 are zoonotic and ruminant‐associated, while ST1, ST5 and ST7 are frequently reported zoonotic STs with broad host ranges.

**Conclusions:**

This study demonstrates a moderate prevalence of *Blastocystis* sp. in ruminants of Qazvin Province, with a diverse set of zoonotic STs including both host‐adapted and common zoonotic STs. These findings align with global prevalence patterns and ST distributions in ruminants, while also complementing recent data from ruminants in Iran. Although the identification of zoonotic STs suggests possible public health implications, definitive evidence of interspecies transmission requires further One Health studies integrating livestock, humans and environmental sources.

## Graphical abstract

Moderate prevalence of *Blastocystis* sp. was detected in cattle, sheep and goats (22.5%) in Qazvin Province. Diverse STs (ST1, ST5, ST7, ST10, ST14) were identified, including both ruminant‐adapted and common human‐associated STs. Females showed higher infection rates than males, highlighting potential host‐related risk factors. Presence of zoonotic STs warrants further One Health studies to investigate cross‐species transmission.

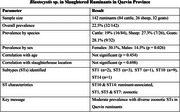

## 1 Introduction

*Blastocystis* sp. is one of the most frequently encountered intestinal protists in both humans and a wide variety of animals, although its clinical importance is still debated (Guyard‐Nicodème et al. 2023; Naguib et al. 2023; Naguib et al. 2024). This stramenopile organism occurs worldwide and has been reported in healthy individuals as well as in patients with gastrointestinal disorders (Aykur et al. 2024). With the development of molecular tools targeting the small subunit ribosomal RNA (SSU rRNA) gene, the remarkable genetic heterogeneity of *Blastocystis* sp. has become evident (Mahdavi et al. 2022). To date, approximately 40–42 distinct subtypes (STs) have been described. Only a limited number of these, particularly ST1–ST4, are consistently found in humans. STs considered to have more zoonotic relevance include ST1–ST7, which have been detected across humans, domestic animals and wildlife, suggesting the possibility of cross‐species transmission. Moreover, STs such as ST8–ST10, ST12, ST14, ST16, ST23, ST35 and ST41 have been reported from both humans and animals, although their epidemiological significance remains less clear (Koehler et al. 2024; Matovelle et al. 2024; Santin et al. 2024; Liu et al. 2025).

In Iran, *Blastocystis* sp. has been the subject of several molecular investigations across both humans and domestic animals, revealing substantial geographical and host‐associated variation. Human studies from Shiraz, Alborz, Ahvaz, Tehran and Kermanshah have consistently shown high colonization rates, often exceeding 9%–55%, with ST1–ST3 as the dominant STs and occasional detection of ST5, ST6, ST7 and ST9 (Salehi et al. 2017; Kataki et al. 2019; Asghari et al. 2020; Sharifi et al. 2020; Sheikh et al. 2020; Karimi et al. 2025; Mohammadi et al. 2025). Animal‐based research has similarly demonstrated considerable heterogeneity: ST10, ST14 and ST5 have frequently been documented in cattle, sheep and goats from regions of Iran (Rostami et al. 2020; Heydarian et al. 2024; Shams et al. 2024), while other zoonotic STs (ST1–ST8) have been identified in livestock, equids, rodents, birds and companion animals (Mohammadpour et al. 2020; Rostami et al. 2020; Sharifi et al. 2020; Asghari, Yousefi, Badali, et al. 2024; Asghari et al. 2024; Bastaminejad et al. 2024; Shams et al. 2024). Collectively, these findings underscore that multiple *Blastocystis* STs circulate within Iranian animal and human populations, although the extent to which livestock contributes to human colonization remains uncertain.

The One Health relevance of *Blastocystis* sp. emerges from the close ecological linkage between humans and livestock in many regions, particularly in low‐ and middle‐income countries where cattle, sheep and goats form integral components of rural livelihoods (Rauff‐Adedotun et al. 2020; Rauff‐Adedotun et al. 2021). These ruminants commonly harbour both host‐adapted STs (e.g., ST10 and ST14) and STs with established zoonotic potential, and therefore may act as reservoirs or amplification hosts (Shams et al. 2021; Shams et al. 2022). Nonetheless, the distribution and diversity of *Blastocystis* sp. STs in Iranian ruminants remain insufficiently characterized, and the degree to which animal‐derived STs overlap with those found in local human populations is not well defined. This gap is particularly notable for provinces such as Qazvin, where livestock production is extensive but molecular epidemiological data are scarce.

In this context, the present study aimed to determine the molecular prevalence and genetic diversity of *Blastocystis* sp. in slaughtered cattle, sheep and goats from Abyek, Buin Zahra and Takestan counties in Qazvin Province, Iran. By characterizing circulating STs and assessing the presence of STs with known or suspected zoonotic relevance, this work provides much‐needed regional baseline data and situates Qazvin within the broader epidemiological landscape of *Blastocystis* sp. research in Iran. The findings contribute to a deeper understanding of *Blastocystis* sp. ecology in ruminants and support ongoing One Health efforts to elucidate potential transmission pathways at the human–animal–environment interface.

## 2 Materials and Methods

### 2.1 Study Area

**FIGURE 1:**
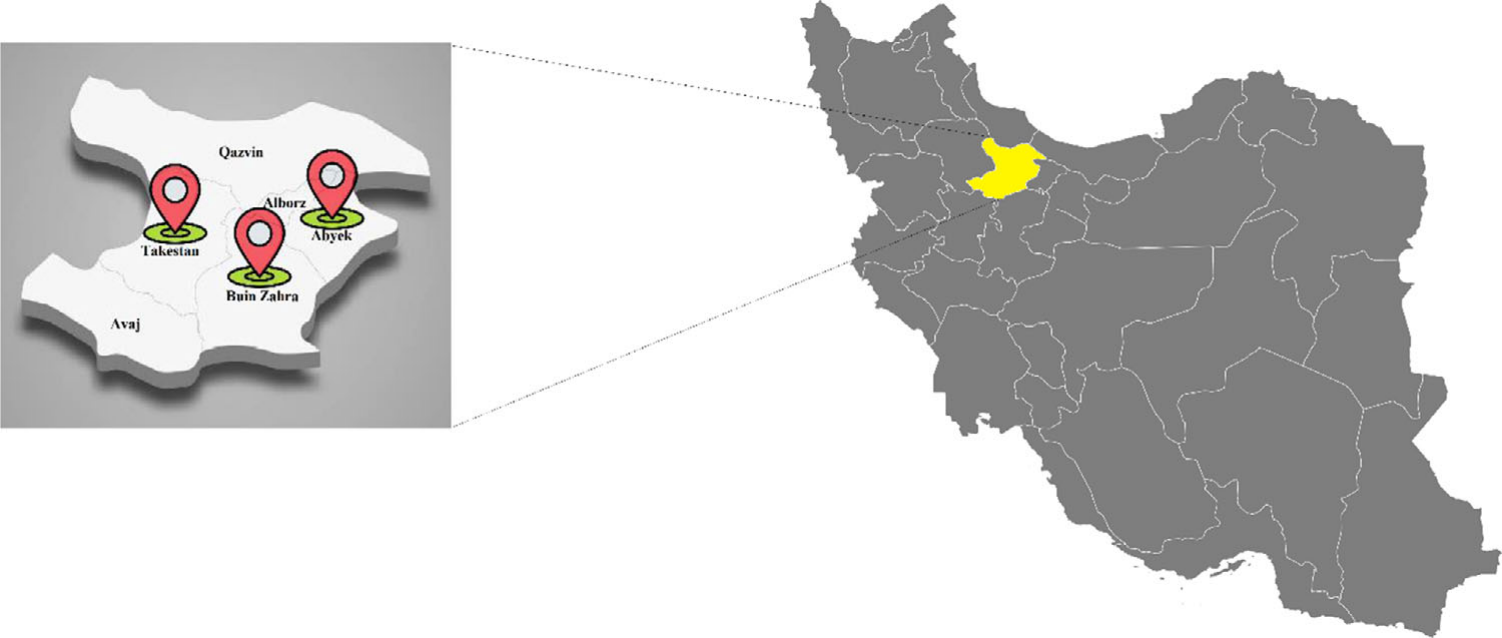
The figure shows the counties in Qazvin Province where ruminant samples were collected from slaughterhouses. The yellow color on the map of Iran indicates Qazvin Province, from which three counties, Abyek, Buin Zahra and Takestan, were surveyed as sampling locations.

This cross‐sectional study was carried out in Qazvin Province, located in the northwest of Iran, in 2025. The province is characterized by a semi‐arid to Mediterranean climate, with cold winters and warm summers, and an annual precipitation that supports both crop cultivation and livestock farming. Animal husbandry represents a major economic activity in this region, with cattle, sheep and goats being raised extensively in rural and peri‐urban areas. Samples were collected from slaughterhouses in three major counties: Abyek, Buin Zahra and Takestan (Figure 1), which together represent key centres of meat production and ruminant farming within the province.

### 2.2 Sample Collection and Processing

A total of 142 fresh faecal samples were collected directly from the rectum of slaughtered animals immediately after evisceration at municipal slaughterhouses, comprising 84 cattle (59.2%), 32 goats (22.5%) and 26 sheep (18.3%). Each sample was placed in a sterile container, labelled with animal species, sex, age group and slaughterhouse location, and transported to the laboratory in insulated boxes with ice packs within 4–6 h of collection. In the laboratory, faecal samples were homogenized and stored at −20°C until further molecular processing.

The exclusive use of slaughterhouse samples was due to logistical and ethical considerations; collecting rectal content post‐slaughter ensured standardized sampling, avoided the need for animal restraint and provided guaranteed access to animals from multiple farms. The total number of samples (*n* = 142) reflects all eligible ruminants available during the study period in the selected slaughterhouses while meeting the minimum statistical threshold for prevalence estimation in a cross‐sectional molecular survey.

### 2.3 DNA Extraction and PCR Amplification

Genomic DNA was extracted from approximately 200 mg of each faecal sample using the QIAamp DNA Stool Mini Kit (Qiagen, Hilden, Germany) according to the manufacturer's instructions. Extracted DNA was eluted in 200 µL of elution buffer and stored at −20°C until downstream analyses. *Blastocystis* sp. was detected in all samples via PCR amplification of a 479 bp SSU rRNA gene fragment, using the primers designed by Santín et al. (2011). The PCR reactions were carried out in a final volume of 25 µL containing 12.5 µL of 2× PCR Master Mix (Ampliqon, Denmark), 1 µL of each primer (10 µM), 2 µL of template DNA and nuclease‐free water to volume. Thermal cycling conditions were as follows: initial denaturation at 95°C for 5 min, followed by 35 cycles of denaturation at 95°C for 30 s, annealing at 58°C for 30 s and extension at 72°C for 1 min, with a final extension at 72°C for 7 min. PCR products were analysed by electrophoresis on 1.5% agarose gels stained with safe stain (Yekta Tajhiz Azma, Iran) and visualized under a UV transilluminator gel documentation system.

### 2.4 Sequencing and Phylogenetic Analysis

Positive PCR products were randomly selected following proportional representation from each host species to avoid species‐biased ST detection. A total of 16 out of 32 positive samples were sequenced, a number determined by budgetary constraints and the high cost of Sanger sequencing, which is a common limitation in molecular epidemiology studies from the region. Sequencing was performed in the forward direction (Pishgam Biotechnology Co., Tehran). Chromatograms were inspected manually and edited using BioEdit v7.2. ST identity was determined through BLAST comparison with GenBank reference sequences. For phylogenetic analysis, sequences were aligned with representative ST sequences and analysed using the Neighbour‐Joining method with the Kimura 2‐parameter model and 1000 bootstrap replicates in MEGA X. *Protomonas lacertae* served as the outgroup. Sequences were deposited in GenBank (accession numbers PX426355‐PX426370). Because single‐direction Sanger sequencing was used, mixed‐ST infections could not be fully assessed.

### 2.5 Statistical Analysis

All data were entered into SPSS software version 26.0 (IBM Corp., Armonk, New York, USA) for statistical analysis. The overall prevalence of *Blastocystis* sp. was calculated as the proportion of PCR‐positive samples among the total examined. Descriptive statistics were used to summarize data by host species, sex and slaughterhouse location. Associations between *Blastocystis* sp. infection status (positive/negative) and categorical variables (animal species, sex, age group and slaughterhouse) were assessed using the chi‐square test of independence. In cases where the expected cell counts were less than five, Fisher's exact test was applied. A *p*‐value of less than 0.05 was considered statistically significant.

## 3 Results

### 3.1 Molecular Prevalence of *Blastocystis* sp. in Ruminants

Out of 142 faecal samples collected from cattle, sheep and goats in slaughterhouses of Abyek, Buin Zahra and Takestan, 32 (22.5%) were positive for *Blastocystis* sp. by PCR. The molecular prevalence by animal species was 19% (16/84) in cattle, 27.3% (7/26) in sheep and 28.1% (9/32) in goats. Prevalence did not differ significantly among the three host species (*χ^2^* = 1.445, df = 2, *p* = 0.486). Analysis by sex revealed that 22 out of 73 females (30.1%) were positive compared with 10 out of 69 males (14.5%). This difference was statistically significant (*χ^2^* = 4.973, df = 1, *p* = 0.026). The prevalence according to slaughterhouse location was 19.5% (8/41) in Abyek, 21.2% (11/52) in Buin Zahra and 26.5% (13/49) in Takestan, with no statistically significant difference among sites (*χ^2^* = 0.720, df = 2, *p* = 0.698). Regarding the influence of age on *Blastocystis* sp. infection, animals aged 1–2 years exhibited the highest prevalence (21/80; 26.3%), followed by those aged 3–5 years (5/20; 25.0%), > 5 years (4/23; 17.4%) and 2–3 years (2/19; 10.5%). However, statistical analysis using the chi‐square test showed no significant association between age group and infection status (*χ^2^* = 2.62, df = 3, *p* = 0.454). The distribution of prevalence across host species, sex, slaughterhouse location and age group is summarized in Table 1.

**TABLE 1:** Molecular prevalence of *Blastocystis* sp. in ruminants of Qazvin province by host species, age, sex and slaughterhouse location.

Variable	Category	Examined (*n*)	Positive *n* (%)	Negative *n* (%)	*χ^2^* (df)	*p*‐value
Age group (Y)	1–2	80	21 (26.3)	59 (73.8)	2.620 (3)	0.454
	2–3	19	2 (10.5)	17 (89.5)		
	3–5	20	5 (25)	15 (75)		
	> 5	23	4 (17.4)	19 (82.6)		
Host species	Cattle	84	16 (19)	68 (81)	1.445 (2)	0.486
	Sheep	26	7 (27.3)	19 (72.7)		
	Goat	32	9 (28.1)	23 (71.9)		
Sex	Female	73	22 (30.1)	51 (69.9)	4.973 (1)	0.026^*^
	Male	69	10 (14.5)	59 (85.5)		
Slaughterhouse	Abyek	41	8 (19.5)	33 (80.5)	0.720 (2)	0.698
	Buin Zahra	52	11 (21.2)	41 (78.8)		
	Takestan	49	13 (26.5)	36 (73.5)		

* Significant at *p* < 0.05.

### 3.2 Distribution of *Blastocystis* sp. STs in Ruminants

**FIGURE 2:**
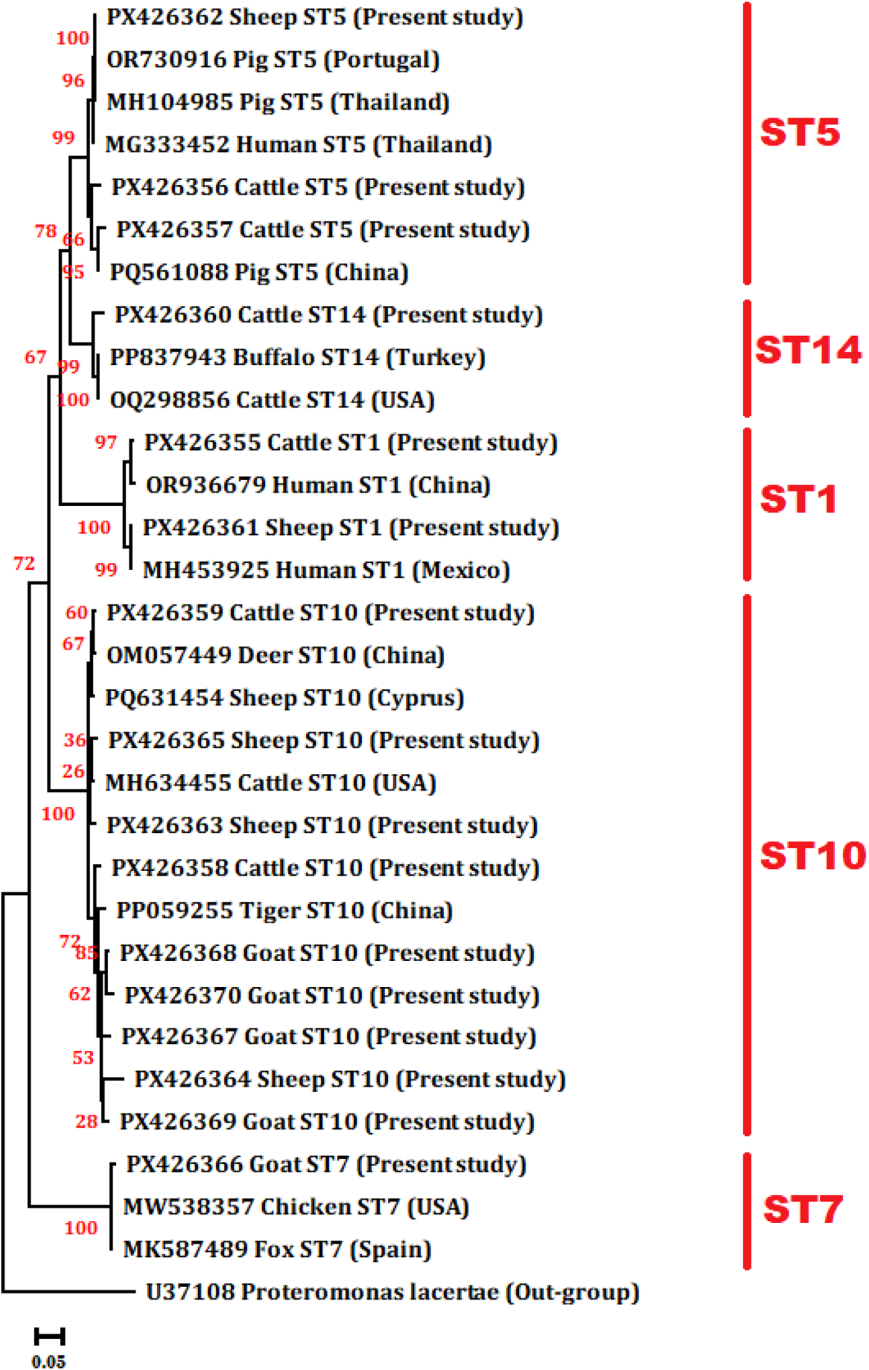
A Neighbour‐Joining phylogenetic tree was constructed in MEGA X (Kimura 2‐parameter model, 1000 bootstraps) to analyse *Blastocystis* sp. isolates and reference sequences from GenBank. The analysis confirmed the isolates clustered into distinct STs, validating ST assignments of Qazvin ruminant isolates relative to global sequences.

Among the 32 PCR‐positive samples, 16 were randomly selected for sequencing. Sequence analysis revealed considerable genetic diversity among *Blastocystis* sp. isolates in ruminants of Qazvin Province, with identification of five zoonotic STs: ST1, ST5, ST7, ST10 and ST14 (Figure 2). In cattle, six isolates were sequenced, including ST1 (*n* = 1), ST5 (*n* = 2), ST10 (*n* = 2) and ST14 (*n* = 1). In sheep, five isolates were sequenced, yielding ST1 (*n* = 1), ST5 (*n* = 1) and ST10 (*n* = 3). In goats, five isolates were sequenced, including ST7 (*n* = 1) and ST10 (*n* = 4). The distribution of STs by host species is presented in Table 2. In the current study, no mixed chromatogram peaks indicative of mixed STs were observed. Moreover, as Sanger sequencing was not performed on all PCR‐positive samples, and because single‐direction sequencing may mask intra‐host variability, the presence of mixed ST infections cannot be ruled out. However, mixed infections could not be definitively identified with the employed primers/sequencing method.

**TABLE 2:** Distribution of *Blastocystis* sp. STs identified in ruminants of Qazvin Province.

Host species	Sequenced samples (*n*)	ST1	ST5	ST7	ST10	ST14
Cattle	6	1	2	—	2	1
Sheep	5	1	1	—	3	—
Goat	5	—	—	1	4	—
Total	16	2	3	1	9	1

## 4 Discussion

The present investigation provides the first molecular data on the occurrence and ST diversity of *Blastocystis* sp. in domestic ruminants of Qazvin Province, Iran. The overall prevalence was 22.5%, with slightly higher infection rates in sheep (27.3%) and goats (28.1%) compared to cattle (19%). These findings fall within the range reported in recent global meta‐analyses, which estimated pooled prevalence at 24.4% for cattle (Shams et al. 2021) and around 20%–25% for small ruminants (Shams et al. 2022). Thus, the prevalence observed in Qazvin reflects the general pattern of moderate infection pressure in livestock worldwide.

When comparing with other Iranian studies, some differences become apparent. In Shahrekord, Southwestern Iran, the overall prevalence in ruminants was lower (14.2%), with strikingly low infection in cattle (0.93%) but higher rates in goats (24.1%) and sheep (17.4%) (Heydarian et al. 2024). In Ilam Province, Western Iran, prevalence was even lower, with only 6% of cattle and 4.7% of sheep testing positive (Shams et al. 2024). By contrast, the present study indicates that *Blastocystis* sp. is more widespread in Qazvin livestock. Differences across provinces may reflect ecological conditions, husbandry practices and diagnostic approaches. Specifically, Qazvin Province has a relatively temperate climate with moderate humidity, which can favour the survival and environmental persistence of *Blastocystis* sp. cysts. In contrast, more extreme climates in some provinces, such as Shahrekord and Ilam may reduce cyst viability. Livestock management practices may also play a role; in Qazvin, mixed‐species grazing, communal watering points, higher stocking densities and traditional husbandry methods can increase contact rates between animals and environmental contamination. Differences in veterinary care, deworming schedules and herd movement patterns may further contribute to regional variations. Together, these ecological and management factors likely explain why *Blastocystis* sp. prevalence is higher in Qazvin compared to other Iranian provinces. In addition, a study on equids in Ardabil Province reported a prevalence of 7.6% overall, with a notable difference between racing (3%) and non‐racing horses (20%), further emphasizing how management and host factors can influence infection rates (Asghari et al. 2024).

Sequencing in the present study revealed five STs (ST1, ST5, ST7, ST10 and ST14), indicating considerable genetic heterogeneity. ST10 was the most frequently detected ST, followed by ST5 and ST1, with ST7 and ST14 also represented. The dominance of ST10, together with the presence of ST14, is consistent with global reviews and with the Shahrekord study, where ST14 accounted for 57.4% of positive isolates and ST10 for 17.1% (Shams et al. 2021; Heydarian et al. 2024). These two STs are regarded as ruminant‐adapted STs, being the most common in cattle and small ruminants worldwide (Rauff‐Adedotun et al. 2021; Shams et al. 2021; Shams et al. 2022; Tavur and Önder 2022), although they have occasionally been recovered from humans (Jinatham et al. 2021). The Ilam study similarly detected ST10 in both cattle and sheep, reinforcing its status as a predominant ruminant ST in Iran (Shams et al. 2024).

Our identification of ST1, ST5 and ST7 is particularly relevant, as these STs are frequently reported in humans and are widely regarded as zoonotic (Paulos et al. 2018; Chen et al. 2023; Wang et al. 2024). In Shahrekord, both ST5 (21.3%) and ST7 (2.1%) were present in ruminants (Heydarian et al. 2024), while in Ilam, ST1 was observed in cattle and sheep alongside human isolates, and ST2 and ST3 were shared between animals and farmers, suggesting potential circulation across host populations (Shams et al. 2024). The equid study in Ardabil also reported ST1, ST2, ST3, ST4, ST7, ST10 and ST14, further illustrating the broad host range of these STs (Asghari et al. 2024). Taken together, these data show that Iranian livestock can harbour both host‐adapted and zoonotic STs.

Of note, all STs identified in our study (ST1, ST5, ST7, ST10 and ST14) are classified as zoonotic. However, their relevance differs. ST1, ST5 and ST7 are frequently found in human populations and have been associated with both asymptomatic carriage and gastrointestinal symptoms (Moosavi et al. 2012; Nagel et al. 2012; El Safadi et al. 2016). In contrast, ST10 and ST14 are most often reported in ruminants and only sporadically in humans (Wang et al. 2018; Rauff‐Adedotun et al. 2023; Yang et al. 2023), where they appear with low diversity and limited frequency. Their occasional detection in people may reflect accidental spillover rather than sustained human‐adapted transmission. Thus, while the presence of ST1, ST5 and ST7 in Qazvin ruminants indicates potential zoonotic importance, ST10 and ST14 likely represent livestock‐adapted STs that rarely establish in humans.

From a One Health perspective, the coexistence of these STs in livestock warrants careful attention. Nevertheless, it must be stressed that the detection of human‐associated STs in animals does not, by itself, prove zoonotic transmission. The Ilam study, which found overlapping STs in animals and their breeders, provides stronger circumstantial evidence of cross‐species circulation (Shams et al. 2024), but definitive demonstration of zoonotic events requires simultaneous human, animal and environmental sampling combined with molecular typing at high resolution. Until such studies are conducted, ruminants should be regarded as possible reservoirs of zoonotic STs rather than confirmed sources of human infection.

Several limitations should be acknowledged. Samples were collected from slaughterhouses, which may not represent the wider population of live herds. Only half of the positive isolates were sequenced, meaning that the true ST spectrum may be even broader. In addition, the lack of paired human and environmental data prevents firm conclusions on zoonotic transmission dynamics. Moreover, this study used single‐direction Sanger sequencing for ST identification, which, although widely applied in molecular parasitology, limits the ability to detect mixed infections involving multiple *Blastocystis* sp. STs within the same host/sample. As a result, coinfections with minor or low‐abundance STs may have remained undetected. Despite these limitations, this study expands current knowledge on *Blastocystis* sp. epidemiology in Iranian livestock. By documenting both predominant ruminant STs (ST10, ST14) and STs with zoonotic potential (ST1, ST5, ST7), our results complement earlier works in Shahrekord, Ilam and Ardabil, and contribute to the growing evidence that ruminants play an important role in the ecology of *Blastocystis* sp. at the human–animal–environment interface.

## 5 Conclusion

This study revealed a moderate prevalence (22.5%) and high genetic diversity of *Blastocystis* sp. in ruminants from Qazvin Province, Iran. Five zoonotic STs were detected (ST1, ST5, ST7, ST10 and ST14), although their significance differs. ST1, ST5 and ST7 are commonly reported from humans and thus have clearer zoonotic potential, while ST10 and ST14 are dominant in ruminants and are only occasionally found in people. The detection of these STs in livestock emphasizes the possibility of cross‐species circulation, but without concurrent human and environmental sampling, zoonotic transmission cannot be confirmed. Our findings underscore the importance of continued molecular surveillance of *Blastocystis* sp. in domestic animals, particularly in regions where humans and livestock live in close contact. Future studies should adopt integrated One Health approaches, incorporating livestock, human and environmental sampling, as well as high‐resolution subtyping, to clarify the epidemiology and zoonotic role of *Blastocystis* sp. Until then, ruminants should be regarded as potential reservoirs of zoonotic STs, highlighting the need for both veterinary vigilance and public health awareness.

## Author Contributions

M.B. and A.A. conceived and designed the study, supervised the project and served as the corresponding authors. F.N.H. and M.M. coordinated sample collection and contributed to fieldwork. A.V.E., F.M. and A.K. performed laboratory experiments, including DNA extraction, PCR amplification and sequencing preparations. M.O. carried out statistical analyses and contributed to data interpretation. M.B., A.A. and D.D. provided expertise in phylogenetic analysis, comparative interpretation of subtype data and critically revised the manuscript for important intellectual content. All authors contributed to drafting or revising the manuscript, approved the final version and agreed to be accountable for all aspects of the work.

## Funding

The authors have nothing to report.

## Ethics Statement

All procedures involving animals were conducted in accordance with ethical standards and approved by the Ethics Committee of Qazvin University of Medical Sciences, Qazvin, Iran (Approval Code: IR.QUMS.REC.1404.072). Faecal samples were collected post‐slaughter, ensuring no direct handling or distress to live animals.

## Conflicts of Interest

The authors declare no conflicts of interest.

## Data Availability

The datasets used and/or analysed during the current study are available in the online version.
